# Characterization of Key Aroma-Active Compounds in Two Types of Peach Spirits Produced by Distillation and Pervaporation by Means of the Sensomics Approach

**DOI:** 10.3390/foods11172598

**Published:** 2022-08-26

**Authors:** Xiaoqin Wang, Wentao Guo, Baoguo Sun, Hehe Li, Fuping Zheng, Jinchen Li, Nan Meng

**Affiliations:** 1 Key Laboratory of Brewing Molecular Engineering of China Light Industry, Beijing Technology and Business University, Beijing 100048, China; 2 Beijing Laboratory for Food Quality and Safety, Beijing Technology and Business University, Beijing 100048, China

**Keywords:** peach spirit, distillation, pervaporation, aroma, sensomics approach

## Abstract

As a deep-processed product of peach, the aroma characteristics of peach spirit have not been systematically studied, and there has been no research on improving the aroma quality through process improvement. Pervaporation technology was used for the first time in the production of peach spirit instead of distillation, and its critical aroma compounds were analyzed compared with distilled peach spirit. Compared to the distilled peach spirit, pervaporation produced peach spirit presented stronger fruity, honey, and acidic aromas, and lighter cooked-apple aroma. Sixty-two and 65 aroma-active regions were identified in the distilled and pervaporation produced peach spirits, and 40 and 43 of them were quantified. The concentrations of esters, lactones, and acids were significantly higher in the pervaporation produced peach spirit than those in the distilled peach spirit, while terpenoids showed opposite tendency. Both of the overall aromas of distilled and pervaporation produced peach spirits were reconstituted successfully by the compounds with OAV ≥ 1. The omission tests identified 10 and 18 compounds as important aroma compounds for distilled and pervaporation-produced peach spirits, respectively. The differences in the key aroma compounds between the two types of peach spirits explained the differences in the aroma profiles.

## 1. Introduction

Peach (*Prunus persica* L.), a typical stone fruit, originated in China and has been cultivated for more than two thousand years [1]. The varieties of peaches are divided into six categories, including sweet, crisp, honey, yellow-fleshed, flat, and nectarine, according to their texture, shape, and skin characteristics [2]. The market time of peaches is mainly from May to September in a vintage, and peaches are often wasted because they are not sold and transported in time [3]. Peach fruit is rich in nutrients, containing essential amino acids, vitamins, organic acids, dietary fiber, folic acid, minerals, and dietary antioxidants. The protein content of peaches is twice that of apples and grapes, and seven times that of pears [3,4,5].

According to the statistics of the 2020 China Fruit Industry Development Report (compiled by the China Fruit Circulation Society), the area and production of peaches in China ranked 1st in the world in 2019, with 89.0 million hm^2^ and 15.993 million tons, respectively [6]. The annual production of peach fruit worldwide is gradually increasing, from 13.2 million tons in 2000 to 24.6 million tons in 2020 [7]. However, the fruits are not storage resistant, dehydration, shriveling, softening, decrease in ascorbic acid content, and decay after harvest [8]. Based on the huge scale of peach fruit cultivation in the world, it is necessary to develop the deep processing industry of peach. Currently, processed products of peach include peach juice [9], jam [10], dried fruit [11], fermented peach wine [1,3], and peach brandy [12]. According to Regulation European Union (EU) 787/2019 legislation, brandy is a spirit drink (alcoholic beverage), made from alcohol, whether or not distillate is added, or distilled at a temperature of less than 94.8% (*v*/*v*), provided that the distillate does not exceed a maximum of 50% of the alcoholic content of the finished product [13]. Peach brandy has been produced in many countries and regions. For example, in California, there is a commercial demand for small amounts of fruit brandy and has occasionally experimented with preparing brandy from fruit, including peach brandy [12]. Peach wine usually has a strong and immediately recognizable peach aroma, with a pleasant taste and smooth aftertaste, while the aroma profile of peach spirit is still unclear [3].

Briefly, spirits are obtained by a process of fermentation, separation, and aging. Among them, the separation technique determines the volatile composition in the spirits, which is very important for the aroma profiles. Distillation, as the most traditional and widely used separation method, is always performed to produce peach brandy [12]. During the distillation process, the extraction and concentration of volatile compounds are achieved by heating. The distillation patterns of volatile compounds are controlled by the boiling point, solubility in alcohol or water, and changes in the alcohol content of the vapor and liquid during the distillation process [14]. However, the distillation process is a step costing 50% of the energy required by a distillery, estimated at 2.77–4.16 kWh per kilogram ethanol produced, accounting for about 3% of the global energy consumption in a year, which causes high production costs and generates greenhouse gas emissions [15]. Pervaporation, a new separation technology, has also been applied in the production of spirits recently. Pervaporation is a membrane technology that separates liquid mixtures by selective transport through non-porous membranes [16]. The application of ceramic α-alumina membranes combined with silk sericin/polyvinylalcohol (PVA) non-porous membrane to treat fermented sugarcane by pervaporation to obtain cachaça was reported by Karp et al. [17]. Sun et al. [18] used a pilot-scale PDMS commercial composite membrane to produce grape spirits by pervaporation, which has better aroma and taste compared to traditional distilled spirits. However, the new separation technique has not been used in the production of peach spirits.

Aroma is not only one of the properties determining the brandy quality, but also an evaluation indicator of the consumer’s preference. Over 500 substances have been detected in spirits, belonging to several compound classes, including esters, alcohols, acids, aldehydes, phenols, terpenoids, and so on [19]. However, there is only a small percentage of compounds interacting with human olfactory receptors to initiate aroma perception [20,21]. Investigating aroma-active substances will facilitate the improvement of spirits processes and enhances the quality of spirits. However, there are a few studies on the aroma analysis of peach brandy or peach spirit. Although Jiang et al. [22] analyzed the volatile compounds in peach brandy extracted by the solid-phase microextraction (SPME) technique, the aroma activity of compounds and their contributions to aroma profile has not been researched. Therefore, to understand the peach spirit comprehensively, it is essential to study the key aroma compounds and characteristic aroma compounds. Besides, to determine the feasibility of producing peach spirit using pervaporation process, the aroma property also needs to be analyzed.

In this study, two types of peach spirits, produced by distillation or pervaporation method, were used as research targets. The present study aims to identify and quantify the aroma-active compounds using gas chromatography-olfactometry (GC-O) and gas chromatography-mass spectrometry (GC-MS); confirm the importance of aroma-active compounds with flavor dilution (FD) factors, odor activity values (OAV), aroma reconstitution tests and omission tests; clarify the key aroma compounds and their influence on the aroma profiles; compare the overall aromas of pervaporation produced peach spirit and distilled peach spirit, and distinguish them by aroma property and critical aroma compounds. The results will help to understand the aroma characterization of peach spirit, and compare the effects of the different separation methods on the aroma of spirits, providing the theoretical foundation to produce high-quality peach spirit.

## 2. Materials and Methods

### 2.1. Description of Samples

**Figure 1:**
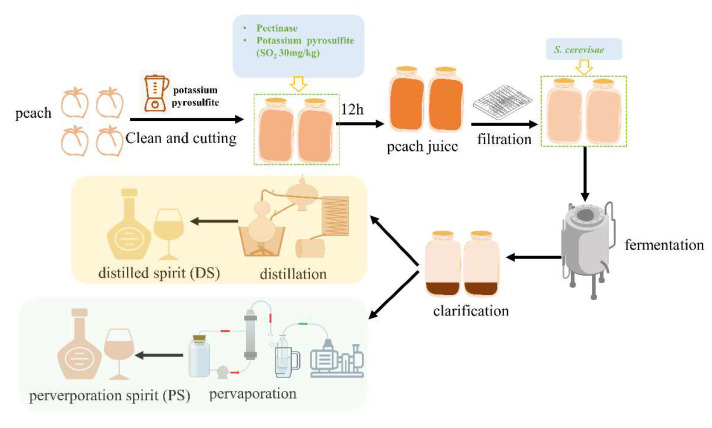
A process flow of DS and PS production.

The peaches used for winemaking were sampled from an orchard in Pinggu District, Beijing, and immediately transferred to the laboratory in Haidian District in October 2021. The peach variety was *Prunus persica* (L.) Batsch cv. Yanhong. The winemaking process is shown in Figure 1. Peaches were cleaned, de-haired, dried, and crushed by mixer (Joyoung, Beijing, China) with potassium metabisulphite to a total SO_2_ level of 30 mg/kg to prevent enzymatic browning and miscellaneous bacteria contamination. A 0.1 g/mL of pectinase ZYM AROM MP (Enartis, Novara, Italy) was added to the peaches and digested at 20 ± 1 °C for 12 h. Twenty liters of clarified juice (total sugar 83 g/L, pH 4.2) were obtained by filtration and transferred into glass fermenters for fermentation. Sugar and tartaric acid (food grade) was added to adjust the total sugar to 240 g/L, and the pH value to 3.5. Then the peach juice was inoculated with 4 g/L Saccharomyces cerevisiae Aroma White yeast (Enartis, Novara, Italy), and controlled the fermentation temperature at 18 ± 2 °C. During the fermentation, the specific gravity and temperature of the fermentation broth were monitored every day. After fermentation, peach wine was ranked three times to clarify. Subsequently, the fermented peach wine was used as raw material to produce peach spirits. Part of the peach wine was distilled twice using a mini-Charente pot still and obtained a spirit with 70% (*v*/*v*) ethanol. Then, the alcohol content was adjusted to 40% with pure water. The final spirit was distillation spirit (DS). The other part of the peach wine was separated using the pervaporation method twice, generating a peach spirit with 40% (*v*/*v*) ethanol, which was named pervaporation spirit (PS). The process condition parameters of pervaporation are described in Supplementary Table S1. The spirits were stored in a cellar with a stable environment (14 °C, 75% relative humidity) for further study.

### 2.2. Chemicals

Absolute ethanol (99.9%), anhydrous sodium sulfate (99.8%), sodium chloride (99.8%), hydrochloric acid (36.0–38.0%) and dichloromethane (HPLC grade, 99.99%) were obtained from Aladdin (Shanghai, China). Ethyl acetate (99.5%), isoamyl acetate (99%), hexyl acetate (99%), ethyl butyrate (99%), ethyl hexanoate (99%), ethyl lactate (99%), ethyl octanoate (99%), ethyl decanoate (99%), ethyl dodecanoate (99%), ethyl tetradecanoate (98%), ethyl oleate (97%), isobutyl acetate (99.5%), ethyl isobutyrate (99%), ethyl isovalerate (99%), 1-butanol (99%), 3-methyl-1-butanol (99%), 1-octanol (99%), leaf alcohol (99%), 3-hydroxy-2-butanone (95%), ethyl 2-furoate (99%), ethyl benzoate (99%), benzyl acetate (98%), phenethyl acetate (99%), phenylethyl alcohol (99%), benzaldehyde (98%), (E,E)-farnesol (96%), linalool (98%), acetic acid (99.7%), 2-methylpropanoic acid (99%), 3-methylbutanoic acid (99%), hexanoic acid (99%), heptanoic acid (98%), decanoic acid (99%), 2-ethylbutyric acid (IS3) (99%), γ-hexalactone (98%), γ-decalactone (98%), γ-octalactone (98%), amyl acetate (99%), 3-methylthiopropanol (98%), hexanal (90%) and benzyl alcohol (99.5%) were obtained from J&K Scientific Ltd. (Beijing, China). Isoamyl lactate (98%), isoamyl decanoate (97%), 3-methylbutyl dodecanoate (97%), ethyl pentadecanoate (97%), ethyl hexadecanoate (95%), isobutanol (99%), 1,1-diethoxyethane (96%), decanal (95%), furfural (98%), γ-nonalactone (97%), 4-heptanol (IS2) (98%), 2-ethyl-1-hexanol (99%), 3-methyl-3-buten-1-ol (98%), citronellol (92%), octanoic acid (98%), and nonanoic acid (98%) were obtained from Tokyo Chemical Industry Co., Ltd. (Tokyo, Japan). 1-hexanol (98%), (E)-β-Damascenone (98%), trans-β-ionone (97%), and p-toluenesulfonic acid n-butyl ester (97%) were purchased from Macklin Biochemical Co., Ltd. (Shanghai, China). trans-nerolidol (95%), and diethyl succinate (99%) were bought from Yuanye Bio-Technology Co., Ltd. (Shanghai, China). In addition, 1-heptanol (99%) was from Aldrich Chemical Co., Inc. (St Louis, MO, USA). 3-Hexenol (97%) was obtained from Alfa Aesar Co., Ltd. (Beijing, China). And γ-butyrolactone (98%) was obtained from Tanmo Quality Assurance Technology Co., Ltd. (Beijing, China). 1-Propanol (99.9%), butyric acid (99%) was bought from Dr. Ehrenstorfer GmbH Co., Ltd. (Augsburg, Germany). Mixtures of n-alkanes C7-C25 (Sigma-Aldrich, Shanghai, China) were used to determine the linear retention indices of the compounds.

### 2.3. Aroma Extract Dilution Analysis with GC-O/GC-MS

#### 2.3.1. Liquid-Liquid Extraction (LLE)

This method referred to Li et al. [23] with modification. Fifty milliliters of spirit were diluted to 10% ethanol by volume with ultrapure water, then saturated with NaCl, and extracted by 50 mL redistilled dichloromethane three times. The organic phase was washed with 50 mL Na_2_CO_3_ solution (0.5 mol/L, pH = 10.0) three times to obtain neutral/basic fraction (NBF). The aqueous phase was acidified by 4.0 mol/L HCl to adjust the pH to 2.0, and extracted by redistilled dichloromethane again to obtain the acidic fraction (AF). Anhydrous Na2SO4 was added to both NBF and AF fractions to remove water, then filtered and concentrated samples to 500 μL using a rotary evaporator. Both NBF and AF samples were stored at −20 °C. One microliter sample was injected into the injector of GC for the analysis of GC-MS and GC-O. For each sample, three parallel experiments were conducted.

#### 2.3.2. Headspace Solid-Phase Microextraction (HS-SPME)

SPME was used to extract volatile compounds to compensate for the lack of highly volatile compounds in the LLE extracted samples. According to the previous method with slight modifications [24]. A SPME fiber (50/30 μm DVB/CAR/PDMS) was used to extract the aroma compounds via HS-SPME. The spirit samples were diluted to 10% ethanol by volume with ultrapure water. Six milliliters of the diluted sample with 3.0 g NaCl were placed into a 20 mL vial capped by a silicon septum tightly. The prepared samples were incubated in a thermostatic bath at 40 °C for 30 min, then inserted a SPME fiber into the vital and extracted at 40 °C for 30 min with stirring at 500 rpm/min. After extraction, the fiber was inserted into the injector of GC to desorb for 8 min followed by GC-O or GC-MS analysis. For each sample, three parallel experiments were conducted.

#### 2.3.3. Aroma Extract Dilution Analysis (AEDA)

AEDA of the concentrates (NBF and AF) obtained by LLE was performed according to the method of Zhao et al. [25]. The original extract by LLE was used as the sample. Either AF or NBF, each concentrated fraction was stepwise diluted 3-fold with dichloromethane as the solvent in a series of 1:3, 1:9, 1:27, …, 1:6561 times. All dilutions were analyzed by GC-O. Referred to Al-Dalali et al. [26] with slight modification, for HS-SPME, AEDA was performed by setting a series of the split ratio at the end of the column, to dilute the sample with helium in a series of 1:3, 1:9, 1:27, …, 1:6561 and analyzed by GC-O. Three trained panelists carried out this research, and each sample was repeated in triplicate by each panelist. The maximum dilution factor that at least two panelists could perceive the odorants is the FD factor.

#### 2.3.4. Conditions of GC-MS/GC-O

GC-MS and GC-O were performed employing an Agilent 7890B GC equipped with an Agilent 5977A MS or a sniffing port (ODP3 C200; Gerstel, Mülheim an der Ruhr, Germany). All the samples were analyzed on a DB-WAX capillary column (60 m × 250 μm i.d., 0.25 μm film thickness; Agilent Technologies Inc, Santa Clara, CA, USA). Helium (99.999%) was used as carrier gas at a constant flow rate of 1.5 mL/min. The injector temperature was 250 °C in splitless mode. The initial temperature of the oven was 40 °C, then raised to 50 °C at a rate of 10 °C/min, and held for 5 min; then raised to 80 °C at 5 °C/min, and held for 5 min; finally raised to 230 °C at 5 °C/min and held for 10 min. The flow split ratio at the end of the column was 1:1 to the MS held at (250 °C) and sniffing-port (230 °C). MS (the electron ionization mode) was in full scan mode with an acquisition range of *m*/*z* 30–350 fragments and ionization energy of 70 eV. The ion source temperature was 230 °C.

#### 2.3.5. Identification of Aroma Compounds

The identification of aroma compounds was achieved by comparing the mass spectrum, linear retention index (RI), and aroma characteristics of the standards or those reported in references. RIs of the compounds detected were calculated by the retention times of a series of n-alkanes (C7-C25). The identified aroma compounds were listed in Table 1.

### 2.4. Quantitation of Aroma Compounds

#### 2.4.1. Direct Injection (DI) Combined with GC-MS

The aromatic compounds, 3-methyl-1-butanol and 1-hexanol were quantified by DI-GC-MS using the internal standard calibration method, according to Wang et al. [27] The sample (990 μL) was spiked with 10.0 μL mixed internal standards (IS1, pentyl acetate 10,000 mg/L; IS2, 4-heptanol 10,000 mg/L; IS3, 2-ethylbutanoic acid, 10,000 mg/L) in a 2.0 mL injection vial, and 1 µL sample was directly injected into the injection port of GC-MS with a 10:1 split ratio. The mass spectrum was chosen as selective ion detection (SIM) mode, and other conditions were the same in Section 2.3.4. Each sample was repeated in triplicate. The mixed standard solutions were prepared as follows. Each standard compound was dissolved in absolute ethanol at high concentrations to prepare the base solution. Then, the standard stock solutions were added to the model solution (40% ethanol by volume, pH 4.0), then diluted into 10 concentrations. The same amount of internal standards was added to the standard solutions. The detection and analysis method was conducted as samples. Detailed information on the calibration curves is listed in Table S2.

#### 2.4.2. LLE Combined with GC-MS

Two hundred and fifty microliters of the internal standard mixture, containing 200 mg/L pentyl acetate (IS1), 200 mg/L 4-heptanol (IS2), and 200 mg/L 2-ethylbutanoic acid (IS3), were added to 30 mL spirit sample. Then, the recombined sample was diluted to 10% ethanol by volume with ultrapure water, saturated with NaCl, and extracted by 50 mL of dichloromethane three times. Anhydrous Na_2_SO_4_ was added to the mixed organic phases overnight to remove water. After filtration, the organic phase was concentrated to 500 µL by spin and nitrogen (99.9999%) blowing as the final sample for detection. One microliter extracted concentrate was submitted to GC-MS with a split ratio of 20:1, and the compounds were quantified using SIM mode. Other conditions were the same with Section 2.3.4. Each sample was conducted in triplicate. The quantitative method was carried out according to Li et al. [23]. The base solutions of standard compounds were diluted in dichloromethane, and other operations were described in Section 2.4.1. Detailed information on the calibration curves is listed in Table S2.

#### 2.4.3. HS-SPME Combined with GC-MS

The aroma compounds, not quantified by DI-GC-MS and LLE-GC-MS, were detected employing HS-SPME combined with GC-MS. Before detection, spirit samples were diluted to 10% ethanol by volume, then 100 μL of the mixed internal standards (IS1, pentyl acetate, 200 mg/L; IS2, 4-heptanol, 200 mg/L; IS3, 2-ethylbutanoic acid, 200 mg/L) was also added. Other operating steps and parameters of HS-SPME and GC-MS were the same as that described in Section 2.3.2 and Section 2.3.4. A synthetic matrix was prepared by mixing the standard stock solution in a 10% (*v*/*v*) ethanol/water solution at pH 4.0, and diluted to ten different concentrations. The internal standards, the same with those in the samples, were also added to the standard solutions. Detailed information on the calibration curves is listed in Table S2.

### 2.5. Sensory Analysis

The sensory panel consisted of 16 trained panelists in the olfactory experiments and quantitative descriptions of aromas, aged 23–26 years, with a male to female ratio of 1:1. All panelists were from the Beijing Key Laboratory of Flavor Chemistry, Beijing Technology and Business University. Before the sensory evaluation experiment, the panelists were trained for 15 min every day for 3 months to improve the accuracy and reproducibility of the test. Sensory tests were performed in a sensory evaluation laboratory at 20 ± 1 °C. First, the panelists described the aroma profile of peach spirits by evaluating the samples, and a list of aroma attributes was obtained based on the frequency and intensity described by the panelists. According to the determined method of Zhu et al. [28], eight sensory attributes were selected, including malty, cooked-apple, fruity, floral, honey, grass, acidic, and alcoholic. Standard solutions of 3-methylbutan-1-ol (malty), (E)-β-damascenone (cooked-apple), ethyl 3-methylbutanoate (fruity), phenylethyl alcohol (floral, honey-like), phenethyl acetate (honey), hexanal (grass), acidic (acetic acid), and alcohol (alcoholic) were used as references in the experiments, respectively [28,29]. Panelists evaluated the similarity of the samples using a 7-point scale. The intensity started at 0 (imperceptible) and increased at 0.5 step each time to 3 (strongly perceptible) [29]. The results gained from 16 evaluators were averaged and plotted as a radar diagram.

### 2.6. Aroma Reconstitution Experiments

In order to reconstitute the original aroma of DS and PS spirit, the aroma active compounds with OAV ≥ 1 were blended in hydroalcoholic solution (40% ethanol by volume, pH 4.0) according to their concentrations quantified in the samples (Table 2). The recombinants were mixed and then balanced for 30 min. The panelists evaluated these reconstituted samples as described in Section 2.5.

### 2.7. Omission Experiments

The omission models were constructed by omitting one or one class of selected aroma compounds from those used in aroma reconstitution experiments. Then each omission model was distinguished by comparison with the corresponding complete reconstituted solution using triangle tests, and panelists need to select the most different one in each group. At last, analyze the frequency that panelists distinguished correctly. Each sample was labeled with a three-digit code randomly, and each test was repeated three times on different dates. The significance of each test was calculated according to the previous reference [30].

### 2.8. Statistical Analysis

The concentrations of aroma compounds were expressed as mean ± standard deviation (SD). OAV was calculated by dividing the concentration of a compound by its odor threshold (Sha et al., 2017). Radar diagrams were carried out using origin 2021 statistical software (OriginLab Corporation, Northampton, MA, USA). The one-way analysis of variance (ANOVA) test was used to analyze the significant difference between two samples using the SPSS 26 software package (SPSS Inc., Chicago, IL, USA). Principal component analysis (PCA) was performed using origin 2021 statistical software. The RIs of aroma compounds were calculated using Qualitative Analysis 10.0 (Agilent Technologies Inc., Santa Clara, CA, USA).

## 3. Results and Discussion

### 3.1. Aroma Profile Analysis of Peach Spirits

**Figure 2:**
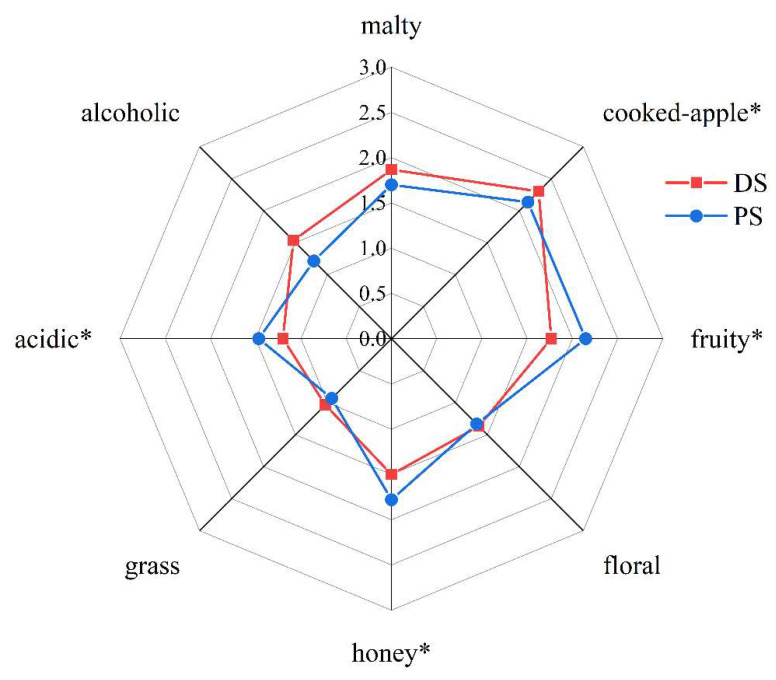
Comparison of aroma profiles of DS and PS. Significance: * *p* < 0.05.

To clarify the differences in the aroma profiles between distilled peach spirit and pervaporation produced peach spirit, sensory analysis was performed. Malty, cooked-apple, fruity, floral, honey, grass, acidic, and alcoholic were chosen as odor descriptors, and the aroma profiles of both peach spirits were extremely different (Figure 2). The scores of cooked-apple property was significantly higher in the distilled peach spirit, while fruity, honey and acidic aroma properties were stronger in the pervaporation produced peach spirit. There was no obvious difference in the intensities of grass, floral, malty, and alcoholic notes. The differences in aroma profiles of distilled peach spirit and pervaporation produced peach spirit were caused by different separation processes.

### 3.2. Identification of Aroma Compounds in Peach Spirits

To comprehensively research the key aroma compounds of peach spirits, and also investigate the odorant compounds responsible for the differences in aroma profiles influenced by two separation processes, samples were extracted by both LLE and SPME, analyzed using AEDA-GC-O and GC-MS, and identified according to RI, standards, aroma and mass spectrum. In distilled peach spirit, a total of 62 odorous regions were detected with FD factors ranging from 1 to 6561, including 22 esters, 9 alcohols, 6 aromatic compounds, 7 terpenes, 1 acetal, 2 carbonyl compounds, 1 lactone, 8 acids, 3 sulfides, and 3 unknown odorants (Table 1). As for the pervaporation produced peach spirit, 65 odor-active regions were detected, including 22 esters, 11 alcohols, 6 aromatic compounds, 5 terpenes, 1 acetal, 4 carbonyl compounds, 5 lactones, 7 acids, 1 sulfide and 3 unidentified odorants. In both peach spirits, the members of esters and alcohols were the most abundant, which was not related to the separation methods, the same as the previous report [31]. There were more terpenoids, while fewer esters, alcohols and lactones in distilled peach spirit than in pervaporation produced peach spirit.

Forty-four compounds with FD factors over or equal to 9 were detected in the distilled peach spirit, while 40 compounds were in the pervaporation produced peach spirit. In both of the spirits, 3-methyl-1-butanol (malty, polish-like), phenylethyl alcohol (dried rose, sweet) and phenethyl acetate (honey, rose-like) had the highest FD values of 6561, 6561, and 2187 respectively, which meant these compounds might be important to the aroma profile of peach spirit regardless of separation methods. 3-Methyl-1-butanol and phenylethyl alcohol were also reported as important aroma compounds in peach wine and peach brandy [22,31]. In the distilled peach spirit, ethyl acetate, isoamyl acetate, ethyl hexanoate, diethyl succinate, 3-methyl-1-butanol, 1-hexanol, 1-heptanol, 1,1-diethoxyethane, furfural, ethyl 2-furoate, benzaldehyde, ethyl benzoate, benzyl acetate, phenethyl acetate, phenylethyl alcohol, citronellol, (E)-β-damascenone, dihydro-β-ionol, trans-nerolidol, trans-β-ionone, (E,E)-farnesol, 3-methylbutanoic acid, nonanoic acid, blackberry thiophenone, 3-methylthiopropanol, and one of the unknown compounds had FD factors over 27. In the pervaporation produced peach spirit, the FD factors of ethyl isovalerate, isoamyl acetate, 1-hexanol, γ-nonalactone, γ-decalactone, benzyl alcohol, (E)-β-damascenone, and 3-methylbutanoic acid were greater than 27. All of them might be the critical aroma compounds for the peach spirits, which should be proved furtherly. The compositional differences between distilled peach spirit and pervaporation produced peach spirit were primarily in esters, lactones, and terpenoids, according to the qualitative results and FD factors of aroma-active compounds.

The numbers of esters in both peach spirits were the same, but the components were different. Ethyl dodecanoate, isoamyl decanoate, ethyl tetradecanoate, ethyl pentadecanoate and ethyl 9-decenoate were only identified as aroma-active compounds in distilled peach spirit, while ethyl isobutyrate, ethyl 2-methylbutyrate, ethyl 3-methyl-butanoate, ethyl 2-hydroxy-3-methyl butyrate and ethyl 3-hydroxybutyrate were identified as aroma-active compounds only in pervaporation produced peach spirit. In peach brandy, ethyl dodecanoate, ethyl tetradecanoate and ethyl 9-decenoate were also identified [22,31]. In addition, the short-chain esters, including ethyl isobutyrate, ethyl isovalerate, and ethyl butanoate, were only detected or with higher FD factors in the pervaporation produced peach spirit compared to distilled peach spirit. While esters with over 14 carbon atoms were mainly detected in the distillation spirit. However, the contribution of esters to the peach spirits produced by different separation methods should be researched deeply.

There were more lactones in the pervaporation produced peach spirit than in the distilled peach spirit. In the distilled peach spirit, only γ-butyrolactone was detected and contributed a fruity note. While γ-butyrolactone, γ-hexalactone (tobacco), γ-octalactone (apricot and peach), γ-decalactone (apricot and peach), and γ-nonalactone (milky notes) were all identified in the pervaporation produced peach spirit. Among them, γ-decalactone and γ-nonalactone showed high FD factors. γ-Decalactone has been reported in the peach brandy [22]. Lactones played an important role in the overall aroma of brandy [32]. In particular, those with 4 to 8 alkyl chains (γ-lactones; γ-C8 to γ-C12) or 3-7 alkyl chains (δ-lactones; δ-C8 to δ-C12) mainly contributed pleasant sensory properties, such as apricot, peach, and coconut, which was similar to the results in this study [33].

Terpenoids have been identified as important contributors to the aroma profile of spirit [34,35]. Contrary to the case of lactones, terpenoids were identified more in distilled peach spirit than in pervaporation produced peach spirit. And citronellol, dihydro-β-ionol, and (E)-β-damascenone exhibit higher FD values in distilled peach spirit compared to pervaporation produced peach spirit. In addition, trans-nerolidol, trans-β-ionone, and (E,E)-farnesol were identified as aroma-active compounds only in the distilled peach spirit.

**Table 1:** Aroma-active compounds identified by GC-O-MS in peach spirits.

No.	Compounds	CAS	Odor Descriptor	Identification ^a^	RI ^b^	FD Factor			
						DS		PS	
						LLE ^c^ (AF/NBF)	SPME ^c^	LLE ^c^ (AF/NBF)	SPME ^c^
**Esters**									
1	Ethyl acetate	141-78-6	Fruity	MS, RI, aroma, S	880	-/81	3	-	9
2	Ethyl isobutyrate	97-62-1	Fruity	MS, RI, aroma, S	878	-	-	-	27
3	Isobutyl acetate	110-19-0	Fruity	MS, RI, aroma, S	900	-	3	-	27
4	Ethyl 2-methylbutyrate	7452-79-1	Fruity	MS, RI, aroma	960	-	-	-/27	9
5	Ethyl isovalerate	108-64-5	Fruity	MS, RI, aroma, S	1103	-	-	-/243	27
6	Ethyl butanoate	105-54-4	Pineapple	MS, RI, aroma, S	1110	-	3	-	27
7	Isoamyl acetate	123-92-2	Banana	MS, RI, aroma, S	1138	-/729	9	-/81	27
8	Ethyl hexanoate	123-66-0	Sweet, fruity	MS, RI, aroma, S	1252	-/243	9	-/27	9
9	Hexyl acetate	142-92-7	Fruity, green	MS, RI, aroma, S	1277	-/3	-	-/9	1
10	Ethyl 2-hexenoate	1552-67-6	Fruity	MS, RI, aroma	1335	-/3	-	-/1	-
11	Ethyl lactate	97-64-3	Fruity	MS, RI, aroma, S	1339	-/9	-	-/1	-
12	Ethyl octanoate	106-32-1	Fruity	MS, RI, aroma, S	1413	-/9	9	-	9
13	Ethyl 2-hydroxyisovalerate	2441-06-7	Fruity	MS, RI, aroma	1422	-	-	1/9	3
14	Ethyl 3-hydroxybutyrate	5405-41-4	Fruity	MS, RI, aroma	1500	-	-	1/-	-
15	Ethyl 2-hydroxy-4-methyl valerate	10348-47-7	Woody, fruity	MS, RI, aroma	1513	-/9	-	-/9	1
16	Isoamyl lactate	19329-89-6	Fruity, nutty	MS, RI, aroma, S	1543	-/3	-	-/3	-
17	Ethyl methyl succinate	-	Fruity	MS, RI	1590	-	-	-/3	-
19	Ethyl decanoate	110-38-3	Fruity	MS, RI, aroma, S	1610	-/9	9	-/3	-
20	Diethyl succinate	123-25-1	Fruity	MS, RI, aroma, S	1645	-/81	-	-/3	-
21	Trimethylene acetate	628-66-0	Green	MS, RI	1672	-	-	1/1	-
22	Ethyl dodecanoate	106-33-2	Floral, sweet	MS, RI, aroma, S	1837	-/9	3	-	-
23	Isoamyl decanoate	2306-91-4	Waxy, fruity	MS, RI, aroma, S	1858	-/27	-	-	-
24	Ethyl tetradecanoate	124-06-1	Sweet	MS, RI, aroma, S	2037	-/9	9	-	-
25	3-Methylbutyl dodecanoate	6309-51-9	Sweet, overripe fruit	MS, RI, aroma, S	2058	-/9	-	-	-
26	Ethyl pentadecanoate	41114-00-5	Sweet, honey	MS, RI, aroma, S	2138	-/27	-	-	-
27	Ethyl hexadecanoate	628-97-7	Waxy, oily	MS, RI, aroma, S	2250	1/9	-	-/3	-
28	Ethyl 9-decenoate	67233-91-4	Unpleasant	MS, RI	2262	-/9	3	-	-
29	Ethyl octadecanoate	111-61-5	Waxy	MS, RI, aroma, S	2449	-/3	-	-/3	-
30	Ethyl oleate	111-62-6	Waxy, oily	MS, RI, aroma, S	2469	-/9	-	-	-
**Alcohols**									
31	1-Propanol	71-23-8	Alcohol	MS, RI, aroma, S	1024	-	9	3/3	-
32	Isobutanol	78-83-1	Burnt	MS, RI, aroma, S	1092	-	-	3/1	81
33	1-Butanol	71-36-3	Fruity	MS, RI, aroma, S	1120	-	-	9/1	3
34	3-Methyl-1-butanol	123-51-3	Malty, nail polish-like	MS, RI, aroma, S	1199	243/6561	81	243/6561	243
35	3-Methyl-3-buten-1-ol	763-32-6	Fruity	MS, RI, aroma, S	1245	-	-	1/3	-
36	1-Pentanol	71-41-0	Fruity, sour	MS, RI, aroma	1258	/3		/1	1
37	4-Methyl-2-pentanol	108-11-2	Almond, toasted	MS, RI, aroma	1300	-/3	-	-	-
38	3-Methyl-1-pentanol	589-35-5	Fruity	MS, RI, aroma, S	1318	-	-	-/1	-
39	1-Hexanol	111-27-3	Green	MS, RI, aroma, S	1346	1/81	81	9/81	9
40	3-Hexenol	544-12-7	Green	MS, RI, aroma, S	1350	-/9	-	3/9	1
41	Leaf alcohol	928-96-1	Green	MS, RI, aroma, S	1365	-/9	-	-	-
42	1-Heptanol	111-70-6	Green	MS, RI, aroma, S	1428	-/81	-	-/3	-
43	2-Ethyl-1-hexanol	104-76-7	Fruity	MS, RI, aroma, S	1495	-	-	-/1	-
44	1-Octanol	111-87-5	Fruity	MS, RI, aroma, S	1529	-/3	-	-	-
**Acetals**									
45	1,1-Diethoxyethane	105-57-7	Sweet, fruity	MS, RI, aroma, S	906	-	27	-	27
**Carbonyl compounds**									
46	3-Hydroxy-2-butanone	513-86-0	Fruity	MS, RI, aroma, S	1285	-	-	9/3	-
47	Furfural	98-01-1	Woody, almond	MS, RI, aroma, S	1419	1/81	-	3/27	-
48	Decanal	112-31-2	Green	MS, RI, aroma, S	1512	-	-	-	3
49	Ethyl 2-furoate	614-99-3	Fruity, burnt	MS, RI, aroma, S	1586	-/81	-	-/1	3
**Lactones**									
50	γ-Butyrolactone	96-48-0	Fruity	MS, RI, aroma, S	1590	-/9	-	-/3	-
51	γ-Hexalactone	695-06-7	Tobacco	MS, RI, aroma, S	1621	-	-	-/9	-
52	γ-Octalactone	104-50-7	Apricot and peach	MS, RI, aroma, S	1810	-	-	-/9	-
53	γ-Nonalactone	104-61-0	Milky notes	MS, RI, aroma, S	1957	-	-	/243	-
54	γ-Decalactone	706-14-9	Apricot and peach	MS, RI, aroma, S	2158	-	-	/729	-
**Aromatic compound**									
55	Benzaldehyde	100-52-7	Almond, woody	MS, RI, aroma, S	1485	-/243	-	-/9	9
56	Ethyl benzoate	93-89-0	Floral, fruity	MS, RI, aroma, S	1639	-/81	3	-/1	3
57	Benzyl acetate	140-11-4	Floral, sweet	MS, RI, aroma, S	1695	-/243	-	-/1	3
58	Phenethyl acetate	103-45-7	Honey	MS, RI, aroma, S	1787	1/2187	9	-/2187	3
59	Benzyl alcohol	100-51-6	Fruit	MS, RI, aroma, S	1843	3/-	-	81/81	27
60	Phenylethyl alcohol	60-12-8	Dried rose	MS, RI, aroma, S	1879	1/6561	3	81/6561	81
**Terpene**									
61	Linalool	78-70-6	Floral	MS, RI, aroma, S	1521	-/9	-	-/9	1
62	Citronellol	106-22-9	Rose-like	MS, RI, aroma, S	1740	-/81	-	9/-	-
63	(E)-β-Damascenone	23696-85-7	Cooked apple	MS, RI, aroma, S	1820	-/2187	3	-/81	81
64	Dihydro-β-ionol	3293-47-8	Woody, floral	MS, RI, aroma	1950	-/243	3	-/3	27
65	trans-Nerolidol	40716-66-3	Floral, tea	MS, RI, aroma, S	2017	-/729	-	-	-
66	trans-β-ionone	79-77-6	Sweet, floral	MS, RI, aroma, S	2065	-/27	-	-	-
67	Eugenol	97-53-0	Sweet	MS, RI, aroma, S	2130	-	-	-/9	-
68	(E,E)-Farnesol	106-28-5	Sweet, floral	MS, RI, aroma, S	2326	-/27	-	-	-
**Acids**									
69	Acetic acid	64-19-7	Acidic	MS, RI, aroma, S	1405	3/-	-	1/-	3
70	2-Methylpropanoic acid	79-31-2	Acidic	MS, RI, aroma, S	1526	3/-	-	9/-	1
71	Butyric acid	107-92-6	Acidic	MS, RI, aroma, S	1598	-	-	9/-	-
72	3-Methylbutanoic acid	503-74-2	Sweat	MS, RI, aroma, S	1629	81/-	-	243/-	27
73	Hexanoic acid	142-62-1	Acidic, cheese	MS, RI, aroma, S	1804	3/-	-	27/-	-
74	Heptanoic acid	111-14-8	Acidic	MS, RI, aroma, S	1913	1/-	-	-	-
75	Octanoic acid	124-07-2	Vegetable, fatty	MS, RI, aroma, S	2021	9/-	1	-	9
76	Nonanoic acid	112-05-0	Coffee	MS, RI, aroma, S	2130	81/-	-	9/-	27
77	Decanoic acid	334-48-5	Fatty	MS, RI, aroma, S	2235	3/-	-	-	-
**Sulfides**									
78	Blackberry thiophenone	13679-85-1	Fruity berry, toasted	MS, RI, aroma	1492	-/2187	-	-	-
79	3-Methylthiopropanol	505-10-2	Cooked potato, roasted	MS, RI, aroma, S	1681	81/729	-	9/9	-
80	p-toluenesulfonic acid n-butyl ester	778-28-9	Unpleasant	MS, RI, aroma, S	1757	-	9	-	-
**Unknown**									
81	Unknown1		Woody	-	1572	-/27	-	-	1
82	Unknown2		Roasted	-	1652	-/3	-	-	
83	Unknown3		Coffee, roasted	-	1960	3/-	-	-	
84	Unknown4		Animal		2063	-	-	-/27	
85	Unknown5		Cheese		2154	-	-	27/-	

^a^ MS, identification of aroma compounds by MS spectra; retention indices (RI), identification of aroma components by calculation of RI on DB-WAX; aroma, aroma compounds were identified by comparison to reference standards by GC-O-MS; S, compounds were identified by pure standards. ^b^ linear retention indexes. ^c^ Method of sample pretreatment. LLE, liquid–liquid extraction; SPME, Solid phase microextraction.

### 3.3. Quantification and OAV Analysis of Aroma Compounds

To further analyze the differences in aroma characteristics between peach spirits produced by distillation or pervaporation, a total of 53 compounds out of 85 compounds identified by GC-O were quantified using either DI-GC-MS, LLE-GC-MS or HS-SPME-GC-MS (Table 2). Other qualitative compounds were not quantified due to a lack of standards or trace concentration. For quantitative analysis, the standard curves of all compounds were constructed using the internal standard method, exhibited good linear correlation coefficients (R² > 0.990), and the relative standard deviation (RSD) of the triplicate samples was ≤15%. Details were shown in Table S2. OAVs were calculated to evaluate the contribution of aroma compounds in the alcoholic beverage matrix (Table 2). A compound is considered to have a contribution to the aroma profile when its OAV is greater than 1. The different OAV and FD values for the same compound are due to its different thresholds in the different matrixes.

Fourteen aroma compounds with OAVs ≥ 1 were found in distilled peach spirit, and 21 aroma compounds with OAVs ≥ 1 were identified in the pervaporation produced peach spirit. In both of the peach spirits, the OAVs of isoamyl acetate, ethyl hexanoate, 3-methyl-1-butanol,1,1-diethoxyethane, and (E)-β-damascenone were relatively higher than other compounds, and their FD factors were also markedly high, which certified the importance of these compounds to the overall aroma of the peach spirit. While phenethyl acetate, phenethyl alcohol and 3-methylbutanoic acid, with obviously high FD factors, had significantly low OAVs, even below 1.

Most esters contribute fruity and floral sensory properties to wines and spirits. The total concentration of all the esters in the pervaporation produced peach spirit was more than in the distilled peach spirit. In the distilled peach spirit, there were 5 esters having greater concentrations than their thresholds, while 8 esters were quantified in the pervaporation produced peach spirit with OAVs ≥ 1. The OAVs of isobutyl acetate, ethyl isovalerate, and ethyl hexanoate in the pervaporation produced peach spirit were significantly higher than those in the distilled peach spirit, especially ethyl hexanoate, which contributed sweet and fruity aroma. Sun et al. [18] reported that compared to the raw wine sample, the contents of ethyl acetate and ethyl hexanoate were concentrated 5.94 times and 1.74 times, respectively, after being separated by the pervaporation process. It indicated pervaporation process was probably positive to concentrate these esters. While ethyl butanoate and ethyl octanoate had higher OAVs in the distilled peach spirit compared to pervaporation produced peach spirit. A similar result was also reported in Cognac produced by Charente pot distillation, that ethyl octanoate and ethyl butanoate had high concentrations and important contributions to brandy [36]. Higher concentrations and OAVs of esters in the pervaporation produced peach spirit explained why the intensity of fruity property was stronger in the pervaporation produced peach spirit than in the distilled peach spirit. As mentioned above, esters preferred to pass through organophilic membranes and be received into the permeate to make spirits, especially short-chain esters with high hydrophobicity, which contribute to fruity scents [37].

Alcohols are mainly generated during the fermentation process. After separation, the total concentration of alcohols in the pervaporation produced peach spirit was significantly higher than that in the distilled peach spirit. Although about 10 alcohols were identified, and 5 and 7 alcohols were quantified in the distilled peach spirit and pervaporation produced peach spirit, respectively, only 3 alcohols had OAVs greater than 1. 3-Methyl-1-butanol (malty and nail polish-like) and 1-hexanol (green) presented OAVs as 7 and 3 in the distilled peach spirit, and as 7 and 6 in the pervaporation produced peach spirit. While isobutanol was only quantified in the pervaporation spirit with the OAV as 22, contributing malty aroma property. Combined with FD factors, the aroma contribution of these three compounds needs attention and further research. Although there were significant differences in the concentrations and OAVs of alcohols in the distilled peach spirit and pervaporation produced peach spirit, the intensities of malty and grass in the two peach spirits had no difference. The reason might be the perceptual interaction of alcohols with other aroma ingredients in a complex matrix environment [38].

Lactones were characteristic aroma compounds of peach and peach wine, contributing to peach and apricot odor. Lactones were mainly concentrated in the pervaporation produced peach spirit. A total of 4 lactones were quantified. Among them, although the FD factor of γ-butyrolactone was low, it was the most abundant lactone in both peach spirits, presenting a fruity aroma, and its content in pervaporation produced peach spirit was 5 times higher than in distilled peach spirit. γ-Decalactone with high FD factor had an OAV as 5 in pervaporation produced peach spirit, presenting apricot and peach aroma. The OAVs of γ-hexalactone and γ-nonanolactone were below 1. Therefore, γ-butyrolactone and γ-decalactone were the main peach-aroma contributors in peach spirit. The differences in lactone concentrations and OAVs between the two peach spirits were in agreement with the typical peach aroma property being stronger in the pervaporation produced peach spirit. Due to high carbon content and the presence of oxygen-containing heterocycles, lactones has high hydrophobicity, which account for the abundance of lactones in pervaporation produced peach spirit [37,39,40]. On the other hand, all these lactones had high boiling points (>200 °C), which resulted in being concentrated hardly by distillation. It implies that producing peach spirit by pervaporation membrane technology was beneficial to retaining the typical aroma of peach.

Terpenoids are important aroma compounds contributing to floral and sweet odor, with relatively low thresholds. Except dihydro-β-ionol, all the terpenoids identified by GC-O were quantified in the two peach spirits. A total of 6 terpenoids were quantified in distilled peach spirit. Among them, citronellol, (E)-β-damascenone, and linalool had OAVs greater than or equal to 1, presenting rose, sweet and floral notes, respectively. Based on the OAV and FD factors, (E)-β-damascenone was potentially the most critical aroma compound in the distilled peach spirit, and it was also suggested as an important aroma compound in Cognac by Uselmann and Schieberle [36]. A total of 3 terpenoids were quantified in pervaporation produced peach spirit, (E)-β-damascenone and eugenol having OAVs greater than 1. A comparative analysis of the terpenoids showed that citronellol and (E)-β-damascenone were more concentrated in the distilled peach spirit, with OAVs 20 times higher than those in the pervaporation produced peach spirit. The above results indicated that the contribution of terpenoids is greater in distilled peach spirit than in pervaporation produced peach spirit. The glycoside-bound form terpenoid is usually 2–8 times that of free form [41]. Acid hydrolysis under mild conditions and continuous heating could liberate volatile compounds from their glycosyl portion during the Charente pot distillation process [42,43]. For example, the heat treatment of diol precursors, as well as the thermal degradation of β-carotene in aqueous media, could form and additionally convert many norisoprenoids [43,44]. Moreover, in the previous research, (E)-β-damascenone has also been proved to be formed during the distillation process, and the concentration of (E)-β-damascenone increased along with the distillation time [45]. Therefore, distillation process is benefit for terpenoids accumulation, while the terpenoid concentration in the pervaporation produced peach spirit was lower due to lack of heating treatment during pervaporation. However, eugenol showed a different accumulation pattern and was only detected in pervaporation produced peach spirit. This might be due to the fact that the chemical structure of eugenol contains a phenyl group, which is more hydrophobic than other terpenoids and facilitates concentrating by pervaporation [37,40].

A total of 7 volatile acids were quantified in the peach spirits, which mainly contributed to the acidic aroma. The concentration of each acid in the pervaporation produced peach spirit was significantly higher than those in the distilled peach spirit, except decanoic acid, which was only detected in the distilled peach spirit. In the distilled peach spirit, the OAVs of all the acids were less than 1. In the pervaporation produced peach spirit, the concentrations of 2-methylpropanoic acid and 3-methylbutanoic acid were the highest among the acids, with OAV of 8 and 4, respectively. The presence of high concentrations of 3-methylbutanol has also been reported in the production of the Brazilian spirit Cachaça using pervaporation [17]. Other acids had extremely low contributions and even made no effect on the overall aroma based on the OAVs. The above results indicated that, compared to other compound classes, acids presented little sensory attribute in the peach spirits. While the comparison between the two peach wines, because the pervaporation process was more conducive to the enrichment of acids, the acidic aroma property was significantly stronger in the pervaporation produced peach spirit. Franitza et al. [46] explained that as acids had higher boiling points than ethanol, losses would occur during the distillation process, while the acids were not affected by the boiling points during the pervaporation.

In the peach spirits, 1,1-diethoxyethane, the only detected acetal compound, had markedly high OAVs with concentrations of more than 4000 μg/L, presenting a sweet sensory attribute. As for furans, furfural was quantified with OAVs of 2 and 3 in the distilled peach spirit and pervaporation produced peach spirit, respectively. Although ethyl 2-furoate was also detected in the distilled peach spirit, the OAV was below 1. Six aromatic compounds with relatively high FD factors were quantified, but all of them had little aroma contribution according to the OAVs, except phenethyl acetate in the distilled peach spirit.

**Table 2:** Quantification parameters, concentration, odor thresholds and OAVs of aroma compounds in peach spirits.

No.	Compounds	Concentrations (μg/L)		Thresholds (μg/L)	OAVs	
		DS	PS		DS	PS
**Esters**						
1	Ethyl acetate ^b^	42,106.30 ± 3614.85	45,362.25 ± 375.58	32,551.6 ^d^	1	1
2	Ethyl isobutyrate ^b^	-	1254.23 ± 8.24	57.47 ^d^	-	21
3	Isobutyl acetate ^b^	Trace	981.85 ± 8.14	922 ^d^	-	1
5	Ethyl isovalerate ^a^	-	32.79 ± 0.04	6.89 ^d^	-	5
6	Ethyl butanoate ^b^	306.77 ± 10.42	165.08 ± 6.85	81.5 ^d^	4	2
7	Isoamyl acetate ^b^	3180.61 ± 113.57	6281.25 ± 60.21	93.93 ^d^	35	67
8	Ethyl hexanoate ^b^	416.76 ± 18.24	6522.65 ± 50.00	55 ^d^	8	127
9	Hexyl acetate ^a^	161.24 ± 3.34	355.67 ± 0.18	1500 ^e^	<1	<1
11	Ethyl lactate ^a^	1222.83 ± 12.33	Trace	128,000 ^d^	<1	-
12	Ethyl octanoate ^a^	533.49 ± 10.49	341.48 ± 1.56	12.87 ^d^	42	27
16	Isoamyl lactate ^a^	13.05 ± 0.58	207.76 ± 0.39	131,703.4 ^d^	<1	<1
19	Ethyl decanoate ^a^	73.12 ± 0.26	5060.85 ± 0.97	1120 ^d^	<1	5
20	Diethyl succinate ^a^	8.63 ± 0.61	1445.60 ± 50	353,193.25 ^d^	<1	<1
22	Ethyl dodecanoate ^a^	150.45 ± 1.36	129.25 ± 4.37	500 ^e^	<1	<1
24	Ethyl tetradecanoate ^a^	96.61 ± 2.70	129.24 ± 4.37	447,068.16 ^f^	<1	-
**Alcohols**						
32	Isobutanol ^c^	-	848,679.75 ± 40,462.05	40,000 ^e^	-	22
33	1-Butanol ^a^	-	983.92 ± 0.39	2733 ^d^	-	<1
34	3-Methyl-1-butanol ^c^	1,133,697.91 ± 30335.64	1,149,273.6 ± 21,513.27	179,000 ^d^	7	7
36	1-Pentanol ^a^	Trace	107.46 ± 0.08	4000 ^e^	-	<1
38	3-Methyl-1-pentanol ^a^	-	120.89 ± 1.25	1000 ^i^	-	<1
39	1-Hexanol ^c^	15,903.45 ± 438.73	32,594.58 ± 1737.89	5370 ^e^	3	6
40	3-Hexenol ^a^	90.68 ± 5.19	Trace	1257 ^h^	<1	-
41	Leaf alcohol ^a^	143.46 ± 12.6	-	1000 ^i^	<1	-
42	1-Heptanol ^a^	149.03 ± 7.54	Trace	26,600 ^e^	<1	-
43	2-Ethyl-1-hexanol ^a^	-	15.44 ± 0.04	1280 ^g^	-	<1
**Acetals**						
45	1,1-Diethoxyethane ^b^	4775.19 ± 85.41	4489.08 ± 62.68	69 ^e^	70	65
**Carbonyl compounds**						
47	Furfural ^a^	166.71 ± 23.15	335.47 ± 0.39	122 ^e^	2	3
49	Ethyl 2-furoate ^a^	30.97 ± 0.37	Trace	16,000 ^g^	<1	-
**Lactones**						
50	γ-Butyrolactone ^a^	84.54 ± 0.30	428.94 ± 0.08	20 ^m^	4	21
51	γ-Hexalactone ^a^	-	116.97 ± 5.00	359,000 ^e^	-	<1
53	γ-Nonanolactone ^a^	-	3.36 ± 0.039	90.66 ^d^	-	<1
54	γ-Decalactone ^a^	-	50.96 ± 0.18	10.87 ^d^	-	5
**Aromatic compounds**						
55	Benzaldehyde ^a^	74.56 ± 1.46	1995.96 ± 1.95	4203.1 ^d^	<1	<1
56	Ethyl benzoate ^a^	109.04 ± 0.64	246.5 ± 0.39	4203 ^d^	<1	<1
57	Benzyl acetate ^a^	39.06 ± 0.27	38.71 ± 1.25	270 ^g^	<1	<1
58	Phenethyl acetate ^a^	3738.40 ± 144.96	359.93 ± 0.39	908.83 ^d^	4	<1
59	Benzyl alcohol ^a^	27.02 ± 3.16	446.02 ± 0.70	40,900 ^e^	<1	<1
60	Phenylethyl alcohol ^a^	1085.98 ± 120.13	10,530.78 ± 622.24	8500 ^d^	<1	1
**Terpenes**						
61	Linalool ^a^	19.67 ± 0.19	Trace	23 ^l^	1	-
62	Citronellol ^a^	372.06 ± 9.17	12.32 ± 0.04	18 ^j^	21	<1
63	(E)-β-damascenone ^a^	626.89 ± 22.22	35.18 ± 0.01	0.4 ^e^	1622	88
65	trans-Nerolidol ^a^	63.36 ± 1.16	-	400 ^j^	<1	-
66	trans-β-ionone ^a^	2.34 ± 0.07	-	4.5 ^j^	<1	-
67	Eugenol ^a^	-	77.72 ± 0.19	21 ^e^	-	4
68	(E,E)-Farnesol ^a^	11.09 ± 1.70	-	1000 ^k^	<1	-
**Acids**						
69	Acetic acid ^a^	179.9 ± 0.52	6827.92 ± 1.95	160,000 ^e^	<1	<1
70	2-Methylpropanoic acid ^a^	292.58 ± 23.08	12,938.45 ± 481.50	1580 ^e^	<1	8
71	Butyric acid ^a^	-	386.28 ± 0.39	964 ^d^	-	<1
72	3-Methylbutanoic acid ^a^	387.43 ± 7.79	4033.45 ± 309.44	1050 ^e^	<1	4
73	Hexanoic acid ^a^	610.95 ± 24.63	1307.33 ± 1.17	2520 ^d^	<1	<1
75	Octanoic acid ^a^	272.16 ± 23.36	1040.49 ± 0.78	2700 ^d^	<1	<1
77	Decanoic acid ^a^	219.86 ± 17.54	-	13,737 ^d^	<1	-
**Sulfides**						
79	3-Methylthiopropanol ^a^	29.51 ± 3.23	406.02 ± 0.39	2110 ^d^	<1	<1

^a^ Quantification by LLE-GC-MS. ^b^ Quantification by SPME-GC-MS. ^c^ Quantification by DI-GC-MS. ^d^ Fan and Xu et al. [47]: 46% ethanol/water (*v*/*v*). ^e^ Liu and Sun et al. [48]: Baijiu. ^f^ Du et al. [49]: 58% ethanol/water (*v*/*v*). ^g^ Van Gemert. [50]: water. ^h^ Uselmann & Schieberle. [36]: water. ^i^ Zea et al. [51]: 18% ethanol/water (*v*/*v*). ^j^ Diéguez et al. [35]: water. ^k^ Li et al. [52]: wine. ^l^ Willner et al. [53]: 40% ethanol/water (*v*/*v*). ^m^ Peinado et al. [54]: 10% (*v*/*v*) ethanol/water (*v*/*v*) adjusted to pH 3.5 with tartaric acid.

### 3.4. Aroma Reconstitution Test

**Figure 3:**
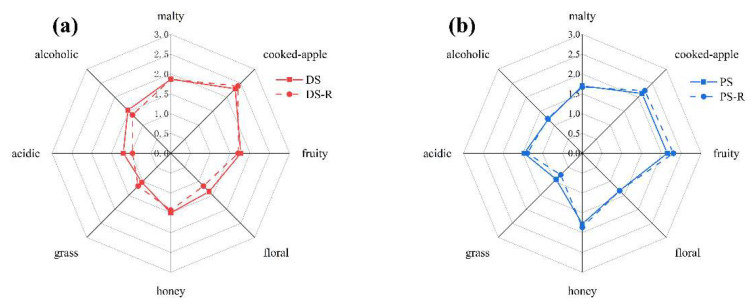
Radar diagram of aroma profiles of original spirit and its reconstitution solution. (**a**) Aroma profiles of DS and its reconstitution. (**b**) Aroma profiles of PS and its reconstitution.

To verify the aroma contribution of odorants with high OAVs to peach spirits, aroma reconstitution tests were carried out. Fourteen odorants with OAV ≥ 1 quantified in distilled peach spirit were dissolved at natural concentrations (Table 2) in 40% ethanol aqueous solution to simulate the original spirit. Similarly, 20 odorants with OAV ≥ 1 quantified in pervaporation produced peach spirit were also dissolved at natural concentrations (Table 2) in another 40% ethanol aqueous solution. Then the reconstituted solutions were evaluated by 16 trained panelists. As shown in Figure 3a, the results of ANOVA indicated that no significant difference existed between the reconstituted solution and distilled peach spirit. The aroma profile of reconstitution was similar to the distilled peach spirit, especially in the honey, malty, fruity, and cooked-apple aroma properties, while the intensities of the alcoholic, floral, and acidic aroma were a little lower than those in the original spirit. As shown in Figure 3b, the aroma profiles of pervaporation produced peach spirit and corresponding reconstitution were nearly overlapped with high similarity of alcoholic, malty, floral, and honey sensory. The intensities of grass and acidic aroma were slightly lower, and the intensities of cooked-apple and fruity were slightly stronger in reconstituted solution compared to the original spirit. There were 5 unknown compounds and several unquantified compounds being unconsidered in the aroma reconstitution test, especially some of them with high FD factors. In addition, the interactions among aroma compounds also influence the aroma profile. These might be the reasons for differentiations presented between the reconstitutions and original spirits.

### 3.5. Omission Test

Omission experiments were performed to investigate the importance of odorants with OAV ≥ 1 on the aroma profiles of distilled peach spirit and pervaporation produced peach spirit. Seventeen omission models and 25 omission models were prepared based on omission of a compound or a class of compounds to compare to the reconstituted models of distilled peach spirit and pervaporation produced peach spirit, respectively (Table 3).

For distilled peach spirit, the absence of all esters was just identified by 9 from 16 panelists (*p* < 0.05). The absence of esters one by one in the omission models was also assessed. The results showed that lack of ethyl hexanoate (1-8) had the most significant effect on the aroma profile (*p* < 0.001), and removing 3-methylbutyl acetate (1-1, *p* < 0.01) or ethyl octanoate (1-5, *p* < 0.05) also had an obvious effect on the overall aroma. However, there was no significant difference when omitted ethyl acetate (1-2, *p* > 0.05) and ethyl butyrate (1-7, *p* > 0.05). Combined with the OAVs, ethyl hexanoate, 3-methylbutyl acetate and ethyl octanoate were critical aroma compounds for distilled peach spirit. And it was speculated that aroma interactions, including masking and inhibition, exist among esters. As for alcohols, when 3-methyl-1-butanol (3-1, *p* < 0.01) and 1-hexanol (3-2, *p* < 0.01) were omitted individually, there was significant difference from the complete reconstitution. In addition, it was more easily to identify the omission solution when both of 3-methyl-1-butanol and 1-hexanol were removed (3, *p* < 0.001). Although the FD factors and OAVs of (E)-β-damascenone and citronellol were markedly high, the mixture models without (E)-β-damascenone (4-1, *p* < 0.05) or citronellol (4-2, *p* < 0.05) was just slightly significantly different in ‘floral’ aroma from the complete recombinant. The same phenomenon was also reported in wine and whisky [55,56,57]. Compared to the aroma properties of complete reconstitution, omissions of 1,1-diethoxyethane (2, *p* < 0.001), γ-butyrolactone (7, *p* < 0.05) or phenethyl acetate (9, *p* < 0.05) was evaluated as a significant difference, while mixture models without 3-methylbutanoic acid (5-1, *p* > 0.05) or furfural (6, *p* > 0.05) had no effect on the aroma.

For pervaporation produced peach spirit, several aroma compounds influenced the aroma profile significantly according to the results of omission tests. The omission models without all esters were significantly different from the complete recombination of pervaporation produced peach spirit. Lack of 3-methylbutyl acetate (1-1, *p* < 0.001), ethyl isobutyrate (1-3, *p* <0.001), ethyl isovalerate (1-4, *p* < 0.001), ethyl octanoate (1-5, *p* < 0.001), or ethyl hexanoate (1-8, *p* <0.001) presented highly significantly lower fruity aroma. While the mixture models absence of ethyl acetate (1-2, *p* > 0.05), ethyl butyrate (1-7, *p* > 0.05) or ethyl decanoate (1-6, *p* < 0.05) could not be recognized easily by panelists in the triangle tests. Compared to distilled peach spirit, the above results indicated esters played a more prominent role in the aroma profile of pervaporation produced peach spirit, contributing to stronger fruity property. It was also easy to be identified by panelists when all alcohols (3, *p* < 0.001), 3-methyl-1-butanol (3-1, *p* < 0.01), 1-hexanol (3-2, *p* < 0.001) or isobutanol (3-3, *p* < 0.01) was removed from the complete reconstitution. Although the OAVs of terpenoids and acids were not high enough, it was proved that they were critical to the aroma profile of pervaporation produced peach spirit. All of the omission solutions without (E)-β-damascenone (4-1, *p* < 0.001), eugenol (4-5, *p* < 0.001), both of the terpenoids (4, *p* < 0.001), 3-methylbutanoic acid (5-1, *p* < 0.001), 2-methylpropanoic acid (5-2, *p* < 0.001), or both of the acids (5, *p* < 0.001) were significantly different from the complete reconstitution, which means the floral and acidic properties were indispensable to the aroma profile of pervaporation produced peach spirit. Lactones have been proved to be key aroma compounds of peach fruit and peach wine [58]. The omission tests results proved this conclusion again. Lack of γ-butyrolactone (7, *p* < 0.001) or γ-decalactone (8, *p* < 0.001) made the intensity of the typical peach aroma decreased. 1,1-Diethoxyethane or furfural omitted also influenced the overall aroma compared to the complete reconstitution. In addition, ethyl isobutyrate, isobutyl acetate, 1,1-diethoxyethane, γ-butyrolactone, (E)-β-damascenone, and 3-methylbutanoic acid were proved to be key active aroma components in peach spirit for the first time.

### 3.6. PCA

**Figure 4:**
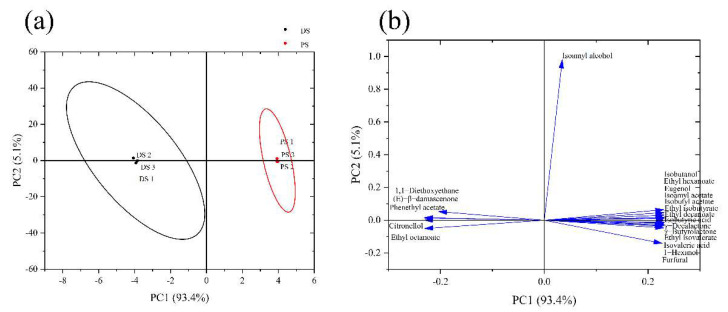
PCA analysis of DS and PS according to the concentrations of the key aroma compounds. (**a**) Score plot of principal component analysis for two peach spirits. (**b**) Loadings plot of principal component analysis for two peach spirits.

To further investigate the key odorants to distinguish the spirits produced by the distillation process of pervaporation process, PCA analysis was performed using the concentrations of all of the key aroma compounds in the peach spirits (Figure 4). The first principal component (PC1) and the second principal component (PC2) explained 93.4% and 5.1%, respectively, indicating these two principal components could represent the original data. Figure 4a showed that PC1 could separate the distilled peach spirit and pervaporation produced peach spirit well, distributed by the negative and positive half-axes, respectively. Ethyl hexanoate, ethyl isobutyrate, isobutyl acetate, 3-methylbutyl acetate, ethyl isovalerate, γ-decalactone, ethyl decanoate, isobutanol, 1-hexanol, γ-butyrolactone, 2-methylpropanoic acid, 3-methylbutanoic acid, eugenol, and furfural were positively corresponding to the aroma characterization of pervaporation produced peach spirit, while 1,1-diethoxyethane, phenethyl acetate, citronellal, (E)-β-damascenone, and ethyl octanoate were highly related to the overall aroma of distilled peach spirit. The distinctive aroma compounds and aroma profiles of peach spirits were formed by different separation methods.

## 4. Conclusions

The aroma profiles and aroma-active compounds of peach spirits produced by distillation and pervaporation were analyzed by sensory analysis, identification, quantification and OAVs, and aroma recombinant and omission tests. The peach spirit produced by distillation showed stronger property of cooked-apple, and the intensities of fruity, honey, and acidic were significantly higher in the peach spirit produced by pervaporation. Using AEDA with GC-O and GC-MS, 62 and 65 aroma-active compounds were identified in distilled peach spirit and pervaporation produced peach spirit, respectively. Among them, 14 and 20 compounds were considered as important contributors to aroma profiles of distilled peach spirit and pervaporation produced peach spirit, respectively, combined with their concentrations and OAVs. Furtherly, both of the aroma profiles of distilled peach spirit and pervaporation produced peach spirit were reconstituted by corresponding important odorants successfully. At last, using omission tests, isoamyl acetate, ethyl hexanoate, 1,1-diethoxyethane, 1-hexanol, (E)-β-damascenone, γ-butyrolactone and phenethyl acetate were proved to be key aroma compounds in the peach spirit produced by distillation, while ethyl isobutyrate, ethyl isovalerate, isoamyl acetate, ethyl hexanoate, ethyl octanoate, 1,1-diethoxyethane, 1-hexanol, (E)-β-damascenone, eugenol, 2-methylpropanoic acid, 3-methylbutanoic acid, γ-butyrolactone and γ-decalactone were the key aroma compounds in the peach spirit produced by pervaporation. Moreover, the peach spirits produced by different separation processes also could be distinguished by the key aroma compounds. This study was the first work to identify the key odor-active volatile compounds in peach spirit through sensory approaches, and to compare the aroma profiles of peach spirits produced by distillation and pervaporation technology systematically. The results are beneficial to understanding the aroma characteristics of peach spirits comprehensively and provide a theoretical foundation for the production of peach spirits and the use of a new separation technique.

## Figures and Tables

**Table 3:** Results of omission experiments from reconstitutions.

NO.	Absence of Compounds	DS		PS	
		N ^a^	Significance ^b^	*n*	Significance
1	All esters	9	*	14	***
1-1	Ethyl acetate	7	-	8	-
1-2	Ethyl isobutyrate	-	-	13	***
1-3	Isobutyl acetate	-	-	10	*
1-4	Ethyl isovalerate	-	-	12	***
1-5	Ethyl butyrate	6	-	8	-
1-6	Isoamyl acetate	11	**	12	***
1-7	Ethyl hexanoate	15	***	13	***
1-8	Ethyl octanoate	9	*	14	***
1-9	Ethyl decanoate	-	-	9	*
2	1,1-Diethoxyethane	13	***	15	***
3	All alcohols	14	***	13	***
3-1	Isobutanol	-	-	10	*
3-2	3-Methyl-1-butanol	10	*	10	*
3-3	1-Hexanol	11	**	12	***
4	All terpenes	8	-	12	***
4-1	Linalool	8	-	-	-
4-2	Citronellol	9	*	-	-
4-3	(E)-β-damascenone	9	*	15	***
4-4	Eugenol	-	-	13	***
5	All acids	-	-	14	***
5-1	2-Methylpropanoic acid	-	-	13	***
5-2	3-Methylbutanoic acid	-	-	14	***
6	Furfural	6	-	14	**
7	γ-Butyrolactone	13	***	13	***
8	γ-Decalactone	-	-	12	***
9	Phenethyl acetate	12	***	-	-
10	Phenethyl alcohol	-	-	7	-

^a^ Number of panelists who identified the aroma difference in the triangle tests correctly. ^b^ Significance: *, 0.05 significant level; **, 0.01 significant level; ***, 0.001 significant level.

## Data Availability

Data are contained within the article or the Supplementary Materials.

## Supplementary Materials

The following supporting information can be downloaded at: https://www.mdpi.com/article/10.3390/foods11172598/s1, Table S1: The process condition parameters of pervaporation membrane in this study; Table S2: Calibration curves for volatile compounds analyzed in this study. Reference [18] is cited in the supplementary materials.
