# Supplementary material for: Characterization of Key Aroma-Active Compounds in Two Types of Peach Spirits Produced by Distillation and Pervaporation by Means of the Sensomics Approach
Source: Foods. 2022 Aug 26;11(17):2598. doi: 10.3390/foods11172598 (PMC9455666; doi:10.3390/foods11172598)
Supplement: Supplementary file 1 [file foods-11-02598-s001.zip › supplementary material.pdf]

Table S1 The process condition parameters of pervaporation membrane in this study

	Support layer	Activate layer
Material[a]	Polyacrylonitrile (PAN) ultrafiltration membrane	Polydimethylsiloxane (PDMS) membrane
Thickness (μm)	30	2
Pore size (nm)	50	-
Membrane area (m^2)	-	0.1

[a] Membrane material information was reported by Sun *et al.* (2020). Operating temperature: 45 °C. Speed of the peristaltic pump: 140 rpm/min.

Table S2 Calibration curves for volatile compounds analyzed in this study.

No.	Compounds	CAS	Method	IS[d]	Quantitative Ion (*m/z*)	Slope	Intercept	R^2	Linear range (μg/L)	LOQ (μg/L)
	**Esters**									
1	Ethyl acetate[b]	141-78-6	SPME-SIM	IS1	74	193.230	-0.046	0.998	1875-75000	＜1875
2	Ethyl isobutyrate[b]	97-62-1	SPME-SIM	IS1	71	5.964	0.004	0.994	9-375	9
3	Isobutyl acetate[b]	110-19-0	SPME-SIM	IS1	56	0.619	-0.048	0.995	7.815-2000	＜7.815
5	Ethyl isovalerate[b]	108-64-5	SPME-SIM	IS1	88	2.755	0.004	0.996	6.24-2000	＜6.24
6	Ethyl butyrate[b]	105-54-4	SPME-SIM	IS1	71	2.722	0.007	0.996	160-10000	＜160
7	Isoamyl acetate[b]	123-92-2	SPME-SIM	IS1	70	1.547	-0.042	0.997	600-30000	＜600
8	Ethyl hexanoate[b]	123-66-0	SPME-SIM	IS1	88	0.245	-0.003	0.999	100-8000	＜100
9	Hexyl acetate[a]	142-92-7	LLE-SIM	IS1	56	1.360	0.011	0.995	4.68-400	4.68
11	Ethyl lactate[a]	97-64-3	LLE-SIM	IS1	75	0.861	-0.049	0.998	11.7-3000	＜11.7
12	Ethyl octanoate[a]	106-32-1	LLE-SIM	IS1	88	0.845	0.032	0.999	14-90000	14
16	Isoamyl lactate[a]	19329-89-6	LLE-SIM	IS1	55	1.136	0.011	0.994	3.63-232	3.63
19	Ethyl decanoate[a]	110-38-3	LLE-SIM	IS1	101	0.172	0.463	0.993	2500-160000	＜2500
19	Ethyl decanoate*[a]	110-38-3	LLE-SIM	IS1	101	0.486	0.005	0.997	3.75-240	3.75
20	Diethyl succinate[a]	123-25-1	LLE-SIM	IS1	101	0.224	0.003	0.993	2.06-260.8	2.06
22	Ethyl dodecanoate[a]	106-33-2	LLE-SIM	IS1	157	1.70	0.107	0.993	92-1500	92
24	Ethyl tetradecanoate[a]	124-06-1	LLE-SIM	IS1	88	0.597	0.027	0.996	30-500	＜30

	**Alcohols**									
32	Isobutanol[c]	78-83-1	DI-SIM	IS2	43	3.440	0.015	0.995	2340-1000000	＜2340
33	1-Butanol[a]	71-36-3	LLE-SIM	IS2	56	1.056	0.008	0.995	3.9-1000	＜3.9
34	Isoamyl alcohol[c]	123-51-3	DI-SIM	IS2	55	2.932	-0.042	0.999	3900-2000000	＜3900
36	1-Pentanol[a]	71-41-0	LLE-SIM	IS2	70	0.694	0.002	0.998	1.4-200	1.4
38	3-Methyl-1-Pentanol[a]	589-35-5	LLE-SIM	IS2	56	0.490	0.002	0.997	12.48-200	12.48
39	1-Hexanol[c]	111-27-3	DI-SIM	IS2	56	2.374	0.008	0.999	780-200000	＜780
40	3-Hexenol[a]	544-12-7	LLE-SIM	IS2	67	0.309	0.019	0.990	7.8-1000	7.8
41	Leaf alcohol[a]	928-96-1	LLE-SIM	IS2	67	2.444	0.052	0.995	12-1600	12
42	1-Heptanol[a]	111-70-6	LLE-SIM	IS2	69	1.994	0.013	0.996	12.5-3200	12.5
43	2-Ethyl-1-hexanol[a]	104-76-7	LLE-SIM	IS2	57	0.424	0.001	0.998	0.71-90	0.71
	**Acetals**									
45	1,1-Diethoxyethane[b]	105-57-7	SPME-SIM	IS1	61	15.127	0.203	0.990	1500-15000	＜1500
	**Carbonyl compounds**									
47	Furfural[a]	98-01-1	LLE-SIM	IS1	67	0.386	0.010	0.999	5.2-1500	5.2
49	Ethyl 2-furoate[a]	614-99-3	LLE-SIM	IS1	95	0.252	0.010	0.994	9.06-580	＜9.06
	**Lactones**									
50	γ-Butyrolactone[a]	96-48-0	LLE-SIM	IS1	56	0.276	0.001	0.988	0.78-200	0.78
51	γ-Hexalactone[a]	695-06-7	LLE-SIM	IS1	85	1.657	0.006	0.998	50-800	＜50
53	γ-Nonanolactone[a]	104-61-0	LLE-SIM	IS1	85	0.512	-0.002	0.996	0.78-100	0.78
54	γ-Decalactone[a]	706-14-9	LLE-SIM	IS1	85	0.368	-0.004	0.999	3.54-300	3.54

	**Aromatic compounds**									
55	Benzaldehyde[a]	100-52-7	LLE-SIM	IS1	105	0.222	0.011	0.993	7.8-5000	＜7.8
56	Ethyl benzoate[a]	93-89-0	LLE-SIM	IS1	105	0.343	0.016	0.993	12.4-400	12.4
57	Benzyl acetate[a]	140-11-4	LLE-SIM	IS1	91	0.350	0.008	0.995	15.2-250	15.2
58	Phenethyl acetate[a]	103-45-7	LLE-SIM	IS1	91	0.674	-0.043	0.997	20.3-5200	20.3
59	Benzyl alcohol[a]	100-51-6	LLE-SIM	IS2	79	0.572	-0.052	0.993	7.0-1800	7
60	Phenylethyl alcohol[a]	60-12-8	LLE-SIM	IS2	122	0.380	-0.421	0.995	70-9000	＜70
	**Terpenes**									
61	Linalool[a]	78-70-6	LLE-SIM	IS2	93	0.525	0.019	0.991	2.6-200	2.6
62	Citronellol[a]	106-22-9	LLE-SIM	IS2	69	0.664	-0.011	0.993	4-500	4
63	*(E)-β*-damascenone[a]	23696-85-7	LLE-SIM	IS1	121	4.665	0.057	0.996	20-720	20
65	*trans*-Nerolidol [a]	40716-66-3	LLE-SIM	IS2	69	0.827	0.049	0.995	25-400	25
66	*trans-β*-ionone[a]	79-77-6	LLE-SIM	IS1	177	0.348	0.001	0.999	0.8-100	0.8
67	Eugenol[a]	97-53-0	LLE-SIM	IS2	149	0.588	-0.007	0.999	3.8-500	＜3.8
68	*(E,E)*-Farnesol[a]	106-28-5	LLE-SIM	IS2	69	1.197	0.003	0.997	2-500	2
	**Acids**									
69	Acetic acid[a]	64-19-7	LLE-SIM	IS3	45	1.646	0.040	0.999	19-5000	＜19
70	2-Methylpropanoic acid*[a]	79-31-2	LLE-SIM	IS3	55	1.907	0.004	0.999	4-500	4
70	2-Methylpropanoic acid[c]	79-31-2	DI-SIM	IS3	55	0.932	0.002	0.997	300-4000	＜300
71	Butyric acid[a]	107-92-6	LLE-SIM	IS3	60	1.107	-0.003	0.999	3.9-1000	3.9
72	Isovaleric acid[a]	503-74-2	LLE-SIM	IS3	60	0.941	0.013	0.997	4-1000	4

72	Isovaleric acid[c]	503-74-2	DI-SIM	IS3	60	1.459	0.001	0.999	312-20000	＜312
73	Hexanoic acid[a]	142-62-1	LLE-SIM	IS3	73	0.784	0.032	0.998	7.8-2000	7.8
75	Octanoic acid[a]	124-07-2	LLE-SIM	IS3	73	0.909	-0.023	0.999	7.8-2000	7.8
77	Decanoic acid[a]	334-48-5	LLE-SIM	IS3	60	1.075	0.078	0.999	18.75-4000	＜18.75
	**Sulfides**									
79	3-Methylthiopropanol[a]	505-10-2	LLE-SIM	IS2	61	2.247	-0.011	0.992	4.2-269.6	＜4.2

[a] Quantified by LLE-SIM. The standard solution was diluted into nine levels successively in CH_2Cl_2. A synthetic matrix (40% ethanol/water, pH = 4.0) was used to establish the calibration curves. [b] Quantified by SPME-SIM. [c] Quantified by DI-SIM. Aroma standards of each level were extracted and analyzed in triplicate under the same conditions as the samples. [d] Corresponding internal standard (IS) of compounds. IS1 indicates pentyl acetate, and IS 2 indicates 4-heptanol, IS 3 indicates 2-ethylbutanoic acid. * represents the standard curve for quantitation of DS. Limit of quantitation (LOQ) of each volatile compounds were caculated based on the signal to noise ratio of 10 obtained by diluting the standard solutions. The LOQs expressed with lower than specific values meant that these compounds were detected with the signal to noise greater than 10 in the last dilution level solution. The linear ranges of each volatile compounds covered their concentrations in two types peach spirits in this study.

References

Sun, X. F., Dang, G. F., Ding, X. B., Shen, C. X., Liu, G. P., Zuo, C. Y., Chen, X. J., Xing, W. H., & Jin, W. Q. (2020). Production of alcohol-free wine and grape spirit by pervaporation membrane technology. Food and Bioproducts Processing, 123, 262-273. http://doi.org/10.1016/j.fbp.2020.07.006.
